# Novel serum biomarkers for predicting neurological outcomes in postcardiac arrest patients treated with targeted temperature management

**DOI:** 10.1186/s13054-023-04400-1

**Published:** 2023-03-16

**Authors:** Hwan Song, Hyo Jin Bang, Yeonho You, Jung Soo Park, Changshin Kang, Hyo Joon Kim, Kyu Nam Park, Sang Hoon Oh, Chun Song Youn

**Affiliations:** 1 Department of Emergency Medicine, Seoul St. Mary’s Hospital, College of Medicine, The Catholic University of Korea, 222 Banpo-daero, Seocho-gu, Seoul, 137-701 Republic of Korea; 2 Department of Emergency Medicine, Chungnam National University Hospital, Daejeon, Republic of Korea

**Keywords:** Cardiac arrest, Outcome, Biomarker, Prognostic accuracy

## Abstract

**Objective:**

To determine the clinical feasibility of novel serum biomarkers in out-of-hospital cardiac arrest (OHCA) patients treated with target temperature management (TTM).

**Methods:**

This study was a prospective observational study conducted on OHCA patients who underwent TTM. We measured conventional biomarkers, neuron‑specific enolase and S100 calcium-binding protein (S-100B), as well as novel biomarkers, including tau protein, neurofilament light chain (NFL), glial fibrillary acidic protein (GFAP), and ubiquitin C-terminal hydrolase-L1 (UCH-L1), at 0, 24, 48, and 72 h after the return of spontaneous circulation identified by SIMOA immunoassay. The primary outcome was poor neurological outcome at 6 months after OHCA.

**Results:**

A total of 100 patients were included in this study from August 2018 to May 2020. Among the included patients, 46 patients had good neurologic outcomes at 6 months after OHCA. All conventional and novel serum biomarkers had the ability to discriminate between the good and poor neurological outcome groups (*p* < 0.001). The area under the curves of the novel serum biomarkers were highest at 72 h after cardiac arrest (CA) (0.906 for Tau, 0.946 for NFL, 0.875 for GFAP, and 0.935 for UCH-L1). The NFL at 72 h after CA had the highest sensitivity (77.1%, 95% CI 59.9–89.6) in predicting poor neurological outcomes while maintaining 100% specificity.

**Conclusion:**

Novel serum biomarkers reliably predicted poor neurological outcomes for patients with OHCA treated with TTM when life-sustaining therapy was not withdrawn. Cutoffs from two large existing studies (TTM and COMACARE substudy) were externally validated in our study. The predictive power of the novel biomarkers was the highest at 72 h after CA.

**Supplementary Information:**

The online version contains supplementary material available at 10.1186/s13054-023-04400-1.

## Introduction

Accurate prognostication remains a crucial factor in the treatment of patients with unconscious postcardiac arrest syndrome (PCAS), primarily focusing on predicting the presence of severe brain damage and poor neurological outcome [1–4]. Identifying patients with poor neurologic outcomes not only avoids unnecessary medical resource consumption but also allows more resources to be focused on patients with recovery potential. Moreover, current postcardiac arrest care guidelines recommend a multimodal approach, as inaccurate outcome prediction can lead to withdrawal of life-sustaining therapy (WLST) in patients with the potential to recover [1, 3]. Clinical examination, electroencephalogram (EEG), somatosensory evoked potentials (SSEP), radiological imaging with computed tomography (CT) or magnetic resonance imaging (MRI) of the brain, and brain injury biomarkers have been used to predict neurological outcomes [1–3, 5, 6]. Among them, biomarkers have the advantage of being easily obtainable from the patient's bedside, providing objective data, and not being affected by sedation [4, 7, 8].

Novel potential biomarkers include the tau protein, neurofilament light chain (NFL), glial fibrillary acidic protein (GFAP), and ubiquitin C-terminal hydrolase-L1 (UCH-L1). The tau protein is a neuroaxonal marker located mainly in the white matter of the central nervous system and is reported to be increased in ischemic stroke and cardiac arrest [9–11]. The NFL, a neuroaxonal marker, is highly accurate in prognostication after cardiac arrest (CA), but it has also been reported to increase in neurodegenerative disorders such as Alzheimer's disease [12–14]. In addition, NFL shows an age-dependent increase in blood [15]. However, the significant increase in NFL levels in the blood suggests acute neuronal injury, such as hypoxic brain injury, rather than chronic neurodegenerative disease [2]. GFAP is an astrocytic marker that is produced as part of a neuroprotective mechanism and has been reported to be associated with prognosis after head trauma, intracerebral hemorrhage, ischemic stroke, and cardiac arrest [16]. UCH-L1 is a neuronal cell body marker that is crucial for neuroaxonal stability and repair after brain injury [2, 17–19]. UCH-L1 is commonly used for the evaluation of traumatic brain injury [2, 18].

The purpose of this study was to examine the usefulness of the abovementioned novel biomarkers in the prognostic evaluation of postcardiac arrest patients who received TTM treatment according to the guidelines. The novel biomarkers were compared with preexisting biomarkers to confirm their clinical feasibility.

## Methods

### Study design and setting

This was a prospective observational study of adult out-of-hospital cardiac arrest (OHCA) patients treated with TTM at two tertiary care academic emergency departments between August 2018 and May 2020: the Department of Emergency Medicine, Seoul St. Mary’s Hospital, The Catholic University of Korea, and the Department of Emergency Medicine, Chungnam Hospital, The Chungnam University of Korea. This study utilized an informed consent form approved by participating hospitals, including the institutional review board (IRB) of Seoul St. Mary’s Hospital (KC18TNSI0396). Written informed consent was obtained from all patients′ legal surrogates and, later, if possible, from the survivors.

The inclusion criteria of this study were as follows: OHCA regardless of etiology of cardiac arrest, age older than 18 years, unconsciousness (Glasgow Coma Scale score < 8) after ROSC and treatment with TTM. The exclusion criteria were as follows: history of cerebrovascular disease, active intracranial bleeding, acute stroke, known severe coagulopathy, cardiac arrest due to trauma or drugs, known limitations in therapy and a do‐not‐attempt resuscitation order, known prearrest cerebral performance category (CPC) 3 or 4, and known terminal disease.

TTM was maintained for 24 h at a temperature of 33–36 °C according to current recommendations and then slowly rewarmed to 36.5 °C at a rate of 0.25 °C per hour [1]. Physicians in charge of patient care and TTM were blinded to the novel biomarker results.

### Novel biomarker analyses

All biomarkers were measured from the patient's blood samples at 0 (after ROSC and before the start of TTM), 24, 48, and 72 h after ROSC. All samples were centrifuged immediately upon collection from the patient and frozen at − 80 °C. Biomarker analyses were performed by investigators blinded to the clinical data. The tau, NFL, GFAP, and UCH-L1 concentrations in the serum samples were measured using the same batch of reagents using a SIMOA neurology 4-plex A kit (PN/102153) in an HD-X immunoassay analyzer (Quanterix Corp, Boston, MA, USA) running ultrasensitive paramagnetic bead-based enzyme-linked immunosorbent assays. Samples were measured at a dilution of 1:4 or, for samples with a very high level, at a dilution of 1:40. All assays were conducted according to the manufacturer’s protocols.

The lower limit of quantification (LLOQ) was 0.964 pg/mL for NFL, 1.87 pg/mL for GFAP, 37.5 pg/mL for UCH-L1, and 0.212 pg/mL for tau. Data were collected using the SIMOA HD-X analyzer using SIMOA HD-X software, version 3.0.2003.04001. The SIMOA immunoassay was performed by DNA Link (DNA Link Inc., Seoul, Korea).

### Outcome assessment

The primary outcome of this study was a poor neurological outcome at 6 months after OHCA, determined by the Cerebral Performance Category (CPC) scale. The CPC scale ranges from 1 to 5 with the following scoring benchmarks: 1 represents good cerebral performance or slight cerebral disability, 2 represents moderate disability or independent activities of daily life, 3 represents severe disability or dependence on others for daily support, 4 represents a comatose or vegetative state, and 5 represents death or brain death. Good neurological outcome was defined as a CPC score of 1 or 2, whereas poor neurological outcome was defined as a CPC score of 3 to 5. Follow-up was performed either face to face or via telephone by independent assessors blinded to the biomarker results.

### Statistical methods

Normality tests were performed for continuous variables, and continuous variables are presented as the means with the standard deviation or as median values with interquartile ranges, as appropriate. Categorical variables are presented as frequencies and percentages. For patient characteristics and comparisons between groups, we used Student's t test or the Mann‒Whitney U test for continuous variables and Fisher's exact test or the chi-square test for categorical variables, and *p* values of ≤ 0.05 were considered statistically significant. The diagnostic performance for poor outcomes was tested with receiver-operating characteristic (ROC) analysis by calculating AUROC. Cut-off values from previous studies were applied to measure the prognostic performance of novel biomarkers to predict poor neurological outcomes after CA [9, 11, 12, 20, 21].

Normal values of novel biomarkers were used to calculate prognostic accuracies for predicting good neurological outcomes after CA. The definitions of normal values were used as they were in previous literature; Tau ≤ 1.55 pg/mL; NFL < 55 pg/mL; GFAP < 22 pg/mL; UCH-L1 < 327 pg/mL [4]. Sensitivity (elevated serum levels in poor outcomes), specificity (normal serum levels in good outcomes), NPV (good outcomes in normal serum levels), and PPV (poor outcomes in elevated serum levels) are presented with 95% confidence intervals.

Statistical analyses were performed using SPSS version 24.0 (SPSS, Chicago, IL, USA) and MedCalc version 15.2.2 (MedCalc Software, Mariakerke, Belgium).

## Results

### Characteristics of study subjects

During the study period, a total of 100 OHCA patients older than 18 years who were treated with TTM were enrolled. Among them, 46 patients had good neurologic outcomes at 6 months after CA. There was no significant difference in the mean age between patients in the good neurologic outcome group (53.3 ± 15.5) and those in the poor neurologic outcome group (58.3 ± 16.2). The comorbidities were not significantly different between the groups. The presumption of cardiac-related arrest, bystander CPR, and initial shockable rhythm were more common in the good neurologic outcome group than in the poor neurological outcome group. Additionally, the time from CA to ROSC was significantly shorter in the good neurological outcome group than in the poor neurological outcome group (24.3 ± 49.9 vs. 54.4 ± 59.69, *p* = 0.015). The time from ROSC to TTM induction was not significantly different between groups (Table 1).

**Table 1:** Baseline characteristics of subjects according to neurological outcomes at 6 months after cardiac arrest

	Good outcome N = 46	Poor outcome N = 54	*P*
Age, years	53.3 ± 15.5	58.3 ± 16.2	0.118
Sex, male	39 (84.8)	39 (72.2)	0.131
Comorbidities			
Hypertension	14 (30.4)	20 (37.0)	0.487
Diabetes mellitus	10 (21.7)	19 (35.2)	0.140
Cardiac cause of arrest	32 (69.6)	18 (33.3)	**< 0.001**
Resuscitation variables			
Witness	32 (69.6)	32 (59.3)	0.285
Bystander CPR	38 (82.6)	34 (63.0)	**0.029**
Initial shockable rhythm	27 (58.7)	9 (16.7)	**< 0.001**
Time from collapse to ROSC, min	24.3 ± 49.9	54.4 ± 59.6	**0.015**
Post-resuscitation variables			
Arterial pH	7.24 ± 0.12	7.16 ± 0.22	**0.029**
Lactate, mmol/L	6.15 ± 3.90	9.69 ± 5.23	**0.001**
TTM induction time, hour	5.0 (2.7, 8.3)	5.0 (3.0, 7.7)	0.922
NSE concentration, ng/mL			
0 h	34.7 (21.5–41.3)	43.0 (29.3–57.4)	**< 0.001**
24 h	22.9 (17.2–33.2)	53.3 (34.2–83.6)	**< 0.001**
48 h	18.0 (13.9–27.3)	50.0 (28.9–19.0)	**< 0.001**
72 h	16.5 (12.3–24.7)	44.9 (26.8–140.4)	**< 0.001**
S100-B concentration, ng/mL			
0 h	0.33 (0.25–0.82)	1.54 (0.74–3.56)	**0.001**
24 h	0.08 (0.05–0.12)	0.29 (0.11–5.18)	**< 0.001**
48 h	0.08 (0.06–0.12)	0.28 (0.09–2.02)	**< 0.001**
72 h	0.07 (0.06–0.13)	0.38 (0.12–1.13)	**< 0.001**

Bold indicates *p*-value less than 0.05

*CPR* cardiopulmonary resuscitation, *ROSC* return of spontaneous circulation, *TTM* targeted temperature management, *NSE* neuron specific enolase, *S100-B* S100 calcium-binding protein

### Serum biomarker levels and neurological outcomes

**Fig. 1:**
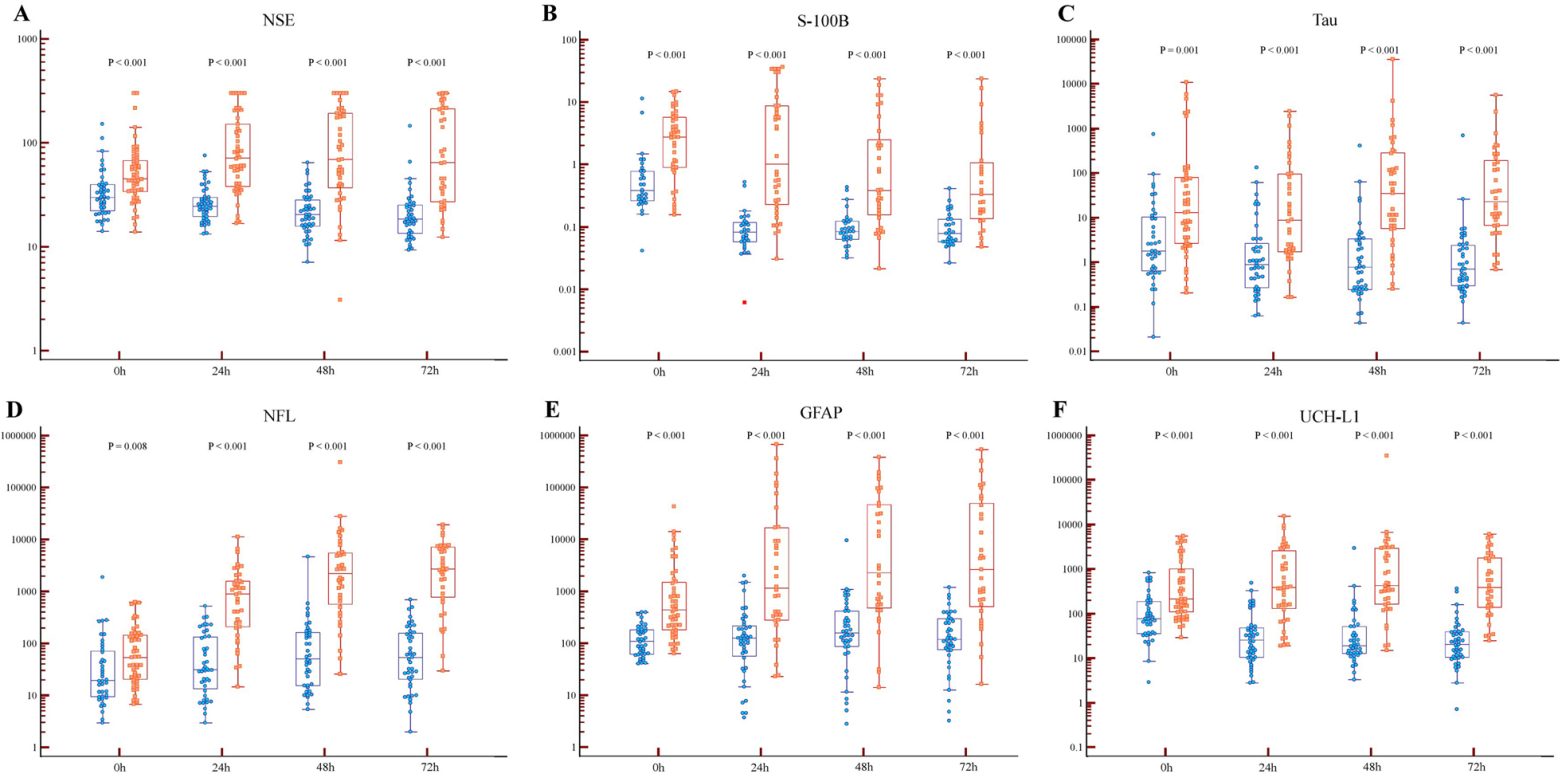
Biomarker concentrations over time according to neurological outcomes. Blue color indicates good neurological outcome group and orange color indicates poor neurological outcome group. Shown are median (line), upper and lower quartiles (box) and range (whiskers). One patient had Tau of 406 pg/mL, NFL of 4,660 pg/mL GFAP of 9520 pg/mL, and UCH-L1 of 2935 pg/mL at 48 h and showed a good outcome at 6 months after CA. Caution is required in interpreting the results. (**A**) NSE levels according to neurological outcome at 0, 24, 48, and 72 hours after CA (**B**) S100-B levels (**C**) Tau levels (**D**) NFL levels (**E**) GFAP levels (**F**) UCH-L1 levels

Median (IQR) serum levels of NSE and S100-B were lower in patients with the good outcome group than in the poor outcome group at all time points. (Table 1, Fig. 1) Median serum levels of novel biomarkers were also lower in patients with the good outcome group than in the poor outcome group at all time points (Table 2, Fig. 1).

**Table 2:** Novel biomarker concentrations at 0, 24, 48 and 72 h after ROSC according to neurological outcomes at 6 months after cardiac arrest

	Good outcome	Poor outcome	*P*
Tau, pg/mL			
0 h	1.78 (0.58–9.79)	6.65 (2.26–32.58)	0.001
24 h	0.99 (0.24–3.29)	3.48 (1.43–42.00)	< 0.001
48 h	0.74 (0.24–2.88)	11.20 (3.12–126.00)	< 0.001
72 h	0.67 (0.28–2.14)	14.00 (4.92–234.25)	< 0.001
NFL, pg/mL			
0 h	19.45 (9.33–78.68)	29.25 (14.30–131.00)	0.008
24 h	31.15 (11.49–169.25)	504.00 (156.50–1540.00)	< 0.001
48 h	51.30 (15.15–165.00)	1515.00 (342.75–4380.00)	< 0.001
72 h	56.65 (15.85–153.25)	2565.00 (429.00–5812.50)	< 0.001
GFAP, pg/mL			
0 h	109.50 (62.20–185.00)	253.50 (134.00–1061.50)	< 0.001
24 h	118.50 (60.23–209.50)	603.00 (234.75–17,050.00)	< 0.001
48 h	143.50 (86.58–327.25)	1138.50 (422.00–48,087.50)	< 0.001
72 h	119.50 (78.70–272.50)	1095.00 (303.00–11,445.00)	< 0.001
UCHL1, pg/mL			
0 h	75.52 (35.19–179.50)	146.85 (73.30–578.78)	< 0.001
24 h	26.53 (10.37–50.58)	309.60 (65.13–1004.55)	< 0.001
48 h	18.85 (12.27–51.63)	324.40 (125.90–1321.98)	< 0.001
72 h	18.40 (9.91–38.26)	222.65 (94.63–858.55)	< 0.001

*NFL* neurofilament light chain, *GFAP *glial fibrillary acidic protein, *UCHL1* ubiquitin C-terminal hydrolase-L1

### Poor outcome prediction

The AUC for predicting the poor outcome of NSE was 0.85 or more at all time points except for immediately after ROSC. The highest AUC for S100-B was 24 h after ROSC (0.901), with a cutoff value of 0.18, a sensitivity of 79.0 (95% CI 62.7–90.4), and a specificity of 93.3% (95% CI 77.9–99.2) (Additional file 1: Table S1).

The AUCs for predicting poor neurological outcome after CA of tau were 0.724, 0.767, 0.837, and 0.906 at 0, 24, 48, and 72 h after ROSC, respectively. (Table 3) The cutoff values for tau with 100% specificity were 748, 131, 406, and 698 pg/mL at 0, 24, 48, and 72 h, respectively, and the sensitivities were 11.1 (95% CI 4.2–22.6), 21.1 (95% CI 9.6–37.3), 18.9 (95% CI 8.0–35.2), and 8.3 (95% CI 1.8–22.5). (Table 4.) The AUCs for predicting poor neurological outcome after CA of NFL, GFAP, and UCH-L1 were highest at 72 h after ROSC. The cutoff values of NFL, GFAP, and UCH-L1 with 100% specificity were 690, 1180, and 356.4 pg/mL at 72 h, respectively, with sensitivities of 77.1 (95% CI 59.9–89.6), 54.6 (95% CI 36.4–71.9), and 50 (95% CI 32.9–67.1), respectively.

**Table 3:** Cutoff values of novel biomarkers with sensitivities, PPVs and NPVs at 0, 24, 48, 72 h after ROSC according to 100% of specificity

	AUC	Cutoff (pg/mL)	Sensitivity	Specificity	PPV	NPV
Tau, 0 h, 100%	0.724	> 748	11.1 (4.2–22.6)	100 (92.3–100.0)	100 (54.1–100.0)	48.9 (38.5–59.5)
Tau, 24 h, 100%	0.767	> 131	21. (9.6–37.3)	100 (92.0–100.0)	100 (63.1–100.0)	59.5 (47.4–70.7)
Tau, 48 h, 100%	0.837	> 406^*^	18.9 (8.0–35.2)	100 (91.6–100.0)	100 (59.0–100.0)	58.3 (46.0–69.9)
Tau, 72 h, 100%	0.906	> 698	8.3 (1.8–22.5)	100 (91.8–100.0)	100 (15.8–100.0)	56.6 (44.7–67.9)
NFL, 0 h, 100%	0.664	> 1890	0 (0.0–7.3)	100 (91.2–100.0)		44.9 (34.4–55.9)
NFL, 24 h, 100%	0.900	> 521	57.9 (40.8–73.7)	100 (92.0–100.0)	100 (84.6–100.0)	73.3 (60.3–83.9)
NFL, 48 h, 100%	0.921	> 4660^*^	36.1 (20.8–53.8)	100 (91.6–100.0)	100 (75.3–100.0)	64.6 (51.8–76.1)
NFL, 72 h, 100%	0.946	> 690	77.1 (59.9–89.6)	100 (91.8–100.0)	100 (87.2–100.0)	84.3 (71.3–93.0)
GFAP, 0 h, 100%	0.850	> 392	53.2 (38.1–67.9)	100 (91.2–100.0)	100 (86.3–100.0)	64.5 (51.3–76.3)
GFAP, 24 h, 100%	0.827	> 1970	44.1 (27.2–62.1)	100 (92.0–100.0)	100 (78.2–100.0)	69.8 (57.0–80.8)
GFAP, 48 h, 100%	0.839	> 9520^*^	43.8 (26.4–62.3)	100 (91.6–100.0)	100 (76.8–100.0)	70 (56.8–81.2)
GFAP, 72 h, 100%	0.875	> 1180	54.6 (36.4–71.9)	100 (91.8–100.0)	100 (81.5–100.0)	74.1 (61.0–84.7)
UCH-L1, 0 h, 100%	0.766	> 827.8	28.6 (16.6–43.3)	100 (91.2––100.0)	100 (76.8–100.0)	53.3 (41.4–64.9)
UCH-L1, 24 h, 100%	0.886	> 492.4	41.0 (25.6–57.9)	100 (92.0–100.0)	100 (79.4–100.0)	65.7 (53.0–76.9)
UCH-L1, 48 h, 100%	0.911	> 2935^*^	27.0 (13.8–44.1)	100 (91.6–100.0)	100 (69.2–100.0)	60.9 (48.3–72.5)
UCH-L1, 72 h, 100%	0.935	> 356.4	50.0 (32.9–67.1)	100 (91.8–100.0)	100 (81.5–100.0)	70.5 (57.4–81.5)

*AUC* area under the curve, *PPV* positive predictive value, *NPV* negative predictive value, *NSE* neuron specific enolase, *S100-B* S100 calcium-binding protein, *NFL* neurofilament light chain, *GFAP* glial fibrillary acidic protein, *UCHL1* ubiquitin C-terminal hydrolase-L1

*Caution with the possibility of laboratory error. Excluding one patient with possible laboratory error, the cutoffs at 48 h were 62.8 for Tau, 582 for NFL, 1880 for GFAP and 415.4 for UCH-L1

**Table 4:** Sensitivity, specificity, PPV, and NPV of a novel biomarker using the cut-off values of previous studies

Biomarkers	Time (h)	Cuttoff value	Sensitivity of original study	Sensitivity (95% CI)	Specificity (95% CI)	PPV (95% CI)	NPV (95% CI)
TTM Trial, 100% specificity							
Tau	24	874.5	4 (2–6)	7.9 (1.7–21.4)	100 (92.0–100)	100	55.7 (53.4–58.0)
	48	148.8	33 (28–38)	27.0 (13.8–44.1)	97.6 (87.4–99.9)	90.9 (57.3–98.7)	60.3 (55.4–65.0)
	72	72.7	42 (36–48)	36.1 (20.8–53.8)	97.7 (87.7–99.9)	92.9 (64.1–99.0)	64.6 (58.7–70.1)
NFL	24	1232	53 (41–64)	36.8 (2.8–54.0)	100 (92.0–100)	100	64.7 (59.0–70.0)
	48	1539	65 (55–74)	58.3 (40.8–74.5)	97.6 (87.4–99.9)	95.5 (74.8–99.3)	73.2 (64.9–80.1)
	72	1756	64 (53–74)	62.9 (44.9–78.5)	100 (91.8–100)	100	76.8 (68.3–83.6)
GFAP	24	3425	17 (13–21)	41.2 (24.7–59.3)	100 (92.0–100)	100	68.8 (62.4–74.5)
	48	2952	19 (15–24)	46.9 (29.1–65.3)	97.6 (87.4–99.9)	93.8 (67.6–99.1)	70.7 (63.5–77.0)
	72	3581	12 (9–17)	48.5 (30.8–66.5)	100 (91.8–100)	100	71.7 (664.5–77.9)
UCH-L1	24	12,175	4 (2–7)	2.6 (0.1–13.5)	100 (92.0–100)	100	53.7 (52.4–54.9)
	48	7945	9 (6–12)	2.7 (0.1–14.2)	100 (91.6–100)	100	53.9 (52.5–55.2)
	72	9170	1 (0–3)	0 (0–9.7)	100 (91.8–100)		54.4 (54.4–54.4)
COMACARE Trial, 99% specificity							
Tau	0	206	0	12.2 (4.6–24.8)	97.5 (86.8–99.9)	85.7 (43.0–98.0)	47.6 (44.7–50.5)
	24	40	21 (8–34)	34.2 (19.6–51.4)	95.5 (84.5–99.4)	86.7 (61.0–96.4)	62.7 (57.0–68.1)
	48	16	75 (61–89)	51.4 (34.4–68.1)	90.5 (77.4–97.3)	82.6 (64.0–92.7)	67.9 (59.9–74.9)
	72	10	88 (77–99)	69.4 (51.9–83.7)	95.4 (84.2–99.4)	92.6 (76.1–98.0)	78.9 (69.4–86.0)
NFL	24	127	78 (65–92)	81.6 (65.7–92.3)	70.5 (54.8–83.2)	70.5 (59.6–79.4)	81.6 (68.8–89.9)
	48	263	83 (71–96)	83.3 (67.2–93.6)	88.1 (74.4–96.0)	85.7 (72.2–93.2)	86.1 (74.7–92.8)
	72	344	85 (73–97)	85.7 (69.7–95.2)	93.0 (80.9–98.5)	90.9 (76.9–96.8)	88.9 (78.0–94.8)
GFAP	0	3330	0	17.0 (7.7–30.8)	100 (91.2–100)	100	50.6 (47.4–53.9)
	24	8018	13 (2–24)	32.4 (17.4–50.5)	100 (92.0–100)	100	65.7 (60.3–70.7)
	48	6262	19 (7–32)	43.8 (26.4–62.3)	97.6 (87.4–99.9)	93.3 (66.0–99.0)	69.5 (62.6–75.7)
	72	4235	29 (14–45)	48.5 (30.8–66.5)	100 (91.8–100)	100	71.7 (64.5–77.9)

*PPV* positive predictive value, *NPV* negative predictive value, *NSE* neuron specific enolase, *S100-B* S100 calcium-binding protein, *NFL* neurofilament light chain, *GFAP* glial fibrillary acidic protein, *UCHL1* ubiquitin C-terminal hydrolase-L1

One patient in our cohort had Tau of 406 pg/mL, NFL of 4,660 pg/mL GFAP of 9520 pg/mL, and UCH-L1 of 2935 pg/mL at 48 h. And this patient had Tau of 0.298 pg/mL, NFL of 22.8 pg/mL GFAP of 22.5 pg/mL, and UCH-L1 of 39.52 pg/mL at 72 h. This patient was a healthy person with no underlying disease. After receiving TTM treatment with VF arrest, he regained consciousness and showed a good outcome at 6 months after CA. There seems to be a possibility of laboratory error rather than a confounder, but the exact reason is unknown. Caution is required in interpreting the results.

When the cutoff values from the TTM trial substudy were applied to this study, the specificity for predicting poor neurological outcomes of the novel biomarkers was 97.6–100%. The sensitivity of Tau was higher in the TTM substudy compared to this study, the sensitivity of NFL was similar to that of this study, and the sensitivity of GFAP was higher in this study compared to the TTM substudy [9, 12, 21].

When the cut-off value of the COMACARE clinical substudy was applied to this study, the specificity of the novel biomarkers except for NFL was 90.5–100%. The sensitivity of Tau was higher in the COMACARE trial substudy than in this study, and the sensitivity of GFAP was higher in this study than in the COMACARE trial substudy. The sensitivity of NFL was similar in the COMACARE clinical trial and in this study, but the specificity was measured at 70.5–93.0% in this study [11, 20].

### Good outcome prediction

Tau levels were within the normal range in 45.0–65.1% of patients with good outcomes (specificity) and elevated above normal levels in 79.0–87.8% of poor outcome patients (sensitivity). (Table 5) Normal Tau levels correctly predicted a good outcome in 75.0–84.9% of patients (NPV). NFL levels were within the normal range in 51.2–72.5% of patients with good outcomes and elevated above normal levels in 46.9–97.1% of poor outcome patients. Normal NFL levels correctly predicted a good outcome in 52.7–95.7% of patients.

**Table 5:** Sensitivity, specificity, PPV, and NPV of a novel biomarker for predicting good neurological outcomes using the cut-off values of predefined normal range

	Sensitivity	Specificity	PPV	NPV
NSE, 0 h	96.3 (87.3–99.6)	4.4 (0.5–15.2)	54.7 (52.7–56.8)	50.0 (2.8–87.2)
NSE, 24 h	98.0 (89.4–99.9)	13.0 (4.9–26.3)	55.1 (52.1–58.0)	85.7 (42.9–98.0)
NSE, 48 h	90.2 (76.9–97.3)	35.6 (21.9–51.2)	56.1 (50.1–61.8)	80.0 (59.3–91.7)
NSE, 72 h	91.7 (77.5–98.3)	36.6 (22.1–53.1)	55.9 (49.7–62.0)	83.3 (61.2–94.1)
S100B, 0 h	100 (91.4–100)	3.3 (0.1–17.2)	58.6 (57.0–60.2)	100
S100B, 24 h	84.9 (68.1–94.9)	63.3 (43.9–80.1)	71.8 (60.9–80.6)	79.2 (61.8–89.9)
S100B, 48 h	78.8 (61.1–91.0)	66.7 (47.2–82.7)	72.2 (60.3–81.6)	74.1 (58.6–85.3)
S100B, 72 h	81.5 (61.9–93.7)	61.3 (42.2–78.2)	64.7 (53.2–74.7)	79.2 (62.2–89.8)
Tau, 0 h	87.8 (75.2–95.4)	45.0 (29.3–61.5)	66.2 (59.2–72.5)	75.0 (56.8–87.2)
Tau, 24 h	79.0 (62.7–90.5)	63.6 (47.8–77.6)	65.2 (55.1–74.1)	77.8 (64.5–87.1)
Tau, 48 h	83.8 (68.0–93.8)	61.9 (45.6–76.4)	66.0 (56.2–74.5)	81.3 (66.7–90.4)
Tau, 72 h	86.1 (70.5–95.3)	65.1 (49.1–79.0)	67.4 (57.4–76.0)	84.9 (70.7–92.9)
NFL, 0 h	46.9 (32.5–61.7)	72.5 (56.1–85.4)	67.7 (53.8–79.0)	52.7 (44.6–60.7)
NFL, 24 h	92.1 (78.6–98.3)	61.4 (45.5–75.6)	67.3 (58.4–75.1)	90.0 (74.8–96.5)
NFL, 48 h	94.4 (81.3–99.3)	52.4 (36.4–68.0)	63.0 (55.1–70.2)	91.7 (73.5–97.8)
NFL, 72 h	97.1 (85.1–99.9)	51.2 (35.5–67.0)	61.8 (54.3–68.9)	95.7 (75.7–99.4)
GFAP, 0 h	100 (92.5–100)	0 (0–8.8)	54.0 (54.0–54.0)	
GFAP, 24 h	100 (89.7–100)	15.9 (6.6–30.1)	47.9 (44.7–51.1)	100
GFAP, 48 h	96.9 (83.8–99.9)	14.3 (5.4–28.5)	46.3 (42.9–49.7)	85.7 (43.2–97.9)
GFAP, 72 h	97.0 (84.2–99.9)	11.6 (3.9–25.1)	45.7 (42.7–48.8)	83.3 (38.0–97.6)
UCH-L1, 0 h	42.9 (28.8–57.8)	87.5 (73.2–95.8)	80.8 (63.5–91.0)	55.6 (48.9–62.1)
UCH-L1, 24 h	56.4 (39.6–72.2)	97.7 (88.0–99.9)	95.7 (75.7–99.4)	71.7 (63.8–78.4)
UCH-L1, 48 h	59.5 (42.1–75.3)	95.2 (83.8–99.4)	91.7 (73.5–97.8)	72.7 (64.2–79.9)
UCH-L1, 72 h	52.8 (35.5–69.6)	97.7 (87.7–99.9)	95.0 (72.8–99.3)	71.2 (66.4–85.9)

*PPV* positive predictive value, *NPV* negative predictive value, *NSE* neuron specific enolase, *S100-B* S100 calcium-binding protein, *NFL* neurofilament light chain, *GFAP* glial fibrillary acidic protein, *UCHL1* ubiquitin C-terminal hydrolase-L1

There were 23 patients with serum levels of Tau within the normal range between 24 and 72 h, and 3 of them had a poor outcome. One patient died during ECMO treatment due to ARDS after mental recovery after TTM, and one patient died due to unrecovered heart failure despite no hypoxic damage on DWI. One patient had hypoxic damage to basal ganglia at DWI but died of status epilepticus. There were 20 patients with serum levels of NFL within the normal range between 24 and 72 h, and only 1 of them had a poor outcome. This patient died of ARDS after mental recovery mentioned above. There were 3 patients with serum levels of GFAP within the normal range between 24 and 72 h, and they all had a good outcome. There were 50 patients with serum levels of UCH-L1 within the normal range between 24 and 72 h, and 10 of them had a poor outcome.

## Discussion

The main finding of this study was that novel biomarkers are a reliable predictor of poor neurological outcomes at 6 months after CA. Cutoffs from two large existing studies (TTM and COMACARE substudy) were applied to this study, resulting in specificity at or close to 100% and sensitivity comparable to the existing studies. This provides clear evidence that these cutoffs are valid for different patient cohorts and laboratories. The AUC for predicting poor neurological outcome was the highest at 72 h after CA for all novel biomarkers. The AUCs for the novel biomarkers were higher than those for conventional biomarkers at 72 h after CA. Among them, the NFL at 72 h after CA had the highest AUC (0.946) and the highest sensitivity (77.1%) with 100% specificity.

Our study had several strengths compared with previous studies [4, 9–12, 16]. First, WLST was not performed on patients included in this study, which minimized the self-fulfilling prophecy. Among the 54 patients with poor neurological outcomes included in this study, 3 (5.6%) patients had a CPC score of 3, and 14 (25.9%) patients had a CPC score of 4, which provided clear evidence that WLST did not influence the results of this study. In addition, since the treating physician was blinded to the novel biomarker results, bias was minimized. Second, this study enrolled patients of Asian ancestry with OHCA with a different etiology than that of previous studies. There were 50 patients (50%) with cardiac-induced OHCA and 36 patients (36%) with initial shockable rhythm. The causes of CA in patients with noncardiac causes were asphyxia in 29 cases, other noncardiac causes in 20 cases, and drug intoxication in 1 case. This contributed to the generalizability of novel biomarkers.

Tau is mainly located in the white matter of the central nervous system and functions to stabilize the structure of microtubules [22]. Mattsson et al. used TTM trial data and reported that tau predicted poor neurological outcome more accurately than NSE at 24 to 72 h after CA and that tau's predictive power increased over time (the AUC at 24, 48, and 72 h was 0.81, 0.90, and 0.91, respectively) [9]. The half-life of tau is approximately 10 h, and a late rise in tau concentrations likely reflects ongoing neuronal damage. In this study, the tau concentration increased over time in the poor neurological outcome group, and the AUC for predicting poor neurological outcome was the highest at 72 h after CA (the AUCs at 24, 48, and 72 h were 0.767, 0.837, and 0.906, respectively). This was consistent with Mattsson's study.

Neurofilaments composed of neurofilament light chains, neurofilament medium chains and neurofilament heavy chains form the cytoskeleton of neurons and are expressed exclusively in neurons [23]. Moseby-Knappe et al. used TTM trial data and reported that the diagnostic performance of NFL was stable from 24 to 72 h (AUC, 0.94), and its performance did not increase significantly when combining serum NFL at different time points [12]. Wihersaari et al. used COMACARE trial data and reported that NFL had excellent prognostic accuracy at 24 h (AUC 0.983) and was a more accurate biomarker for prognostication after CA than NSE [20]. However, in this study, the prognostic power of NFL was the highest at 72 h, contrary to previous studies. One possible explanation is due to differences in the patients included in the study. Both the TTM trial and COMACARE trial included only patients with cardiac causes of cardiac arrest and the patient population was different from that in this study in several aspects, such as the rate of witnessed arrest and total anoxic time. Additionally, this study only included 100 patients, which is a small number compared to the number of patients included in the study by Moseby-Knappe et al.

GFAP is a structural component of intermediate filaments in the astrocyte cytoskeleton, and GFAP production is upregulated after ischemia, which is considered a neuroprotective mechanism [24]. The accuracy of GFAP in predicting neurological outcomes after CA appears to be better at 48 and 72 h after CA compared to earlier time points, with AUC values reported between 0.65 and 0.89, respectively [16, 25, 26]. Ebner et al. used TTM trial data, and thresholds with 100% specificity for predicting poor neurological outcome at 24–72 h after CA were reported as 3425, 2952, and 3581 pg/mL, with sensitivities of 17, 19, and 12%, respectively [21]. On the other hand, thresholds with 100% specificity for predicting poor neurological outcome at 24–72 h after CA were 1970, 9520, and 1180 pg/mL with sensitivities of 44, 44, and 55%, respectively, in this study. However, the cutoff of 9520 pg/mL at 48 h has the potential for laboratory error that requires careful interpretation. Excluding this patient, the cutoff at 48 h is 1880 pg/mL. The reason for the higher sensitivity in this study compared to that in previous studies is difficult to explain, suggesting that more research is needed.

UCH-L1 is a very abundant protein in the brain and consists of 223 amino acids. Ebner et al., using TTM trial data, reported that UCH-L1 had good accuracy in predicting poor neurological outcome after CA (AUC between 0.85 and 0.87). UCH-L1 was significantly better at predicting poor neurological outcome after CA than NSE at 24 and 48 h but not at 72 h. This was explained by the shorter half-life of UCH-L1 compared to that of NSE [21]. However, in this study, the prognostic power of UCH-L1 was the highest at 72 h, which differed from a previous study. As mentioned previously, the TTM trial only included patients with cardiac-caused cardiac arrest and the patient population was different from this study in several aspects, such as the rate of witnessed arrest and total anoxic time. In other words, it is possible that the results differed due to differences in disease severity.

The Additional file 1: Table S2 presents the *p* value of the De-long test comparing the AUC curves of novel biomarkers and conventional biomarkers for predicting poor neurological outcomes after CA. The AUC of GFAP was superior to that of NSE and S100B in predicting poor neurological outcomes after CA at 0 h (*p* = 0.0307 for NSE and *p* = 0.0029 for S100B), and the AUC of NFL and UCH-L1 were superior to S100B at 48 h (*p* = 0.0333 for NFL and *p* = 0.0135 for UCH-L1). Both NFL and UCH-L1 predicted poor neurological outcomes after CA better than NSE and S100-B at 72 h (*p* = 0.0168 for NFL vs NSE, *p* = 0.0148 for NFL vs S100-B, *p* = 0.0361 for UCH-L1 vs NSE and *p* = 0.0069 for UCH-L1 vs S100B). These results comparing the novel biomarker with the conventional biomarker require further validation.

Predicting a good outcome is as important clinically as predicting a poor outcome in comatose PCAS patients. In this study, the biomarkers within the normal range at 24 h showed good neurological outcome at 71.7–100%, and the biomarkers within the normal range at 24–72 h showed good neurological outcome at 80–100%., which is consistent with the results of the TTM trial substudy [4]. Especially, patients with NFL or GFAP levels within the normal range do not have hypoxic-ischemic encephalopathy (HIE) with a very high probability. This provides evidence for preventing erroneous WLST and providing more critical resources to patients with good outcomes.

There were 29 patients with an NFL level of 500 pg/mL or higher at 72 h, and 28 of them had a poor outcome. There were 25 patients with an NFL level of 1000 pg/mL or higher, and all of them had poor outcomes. Therefore, patients with an NFL of 1000 pg/mL at 72 h have a very high probability of having HIE.

Our study had several limitations. First, this was an observational study with a small sample size, which raises the concern of type II errors. In addition, the comparison of prognostic ability between biomarkers was not possible due to the small sample size. Second, although there was no WLST in this study, 11 patients died before 72 h. Missing data caused by early deaths may have increased the possibility of bias. Third, the analysis methods for novel biomarkers are not standardized. We used the highly sensitive SIMOA immunoassay, which is not available in most laboratories. In addition, it is impossible to compare our results with previous studies that did not use this method. Forth, due to the nature of observational studies, the results of our analysis and the cutoff values for novel biomarkers require further validation. Finally, we could not prove whether the novel biomarkers could be more useful in clinical practice than the conventional biomarkers. Several modalities are used to predict neurological outcomes in patients with CA, and the current guidelines recommend using a combination of several modalities. Therefore, the clinical usefulness of the novel biomarker must be demonstrated in a combination of several modalities suggested in the current guidelines.

## Conclusions

Novel serum biomarkers predicted poor neurological outcome after CA with high accuracy. Cutoffs from two large existing studies (TTM and COMACARE substudy) were externally validated in our study. The predictive power of novel biomarkers was the highest at 72 h after CA.

## Supplementary Information

**Additional file 1.** Additional statistical analysis of biomarkers for predicting poor neurological outcomes and comparison of novel and conventional biomarkers for predicting poor neurological outcomes.

## Data Availability

The datasets used and/or analyzed during the current study are available from the corresponding author on reasonable request.
