# Supplementary material for: Novel serum biomarkers for predicting neurological outcomes in postcardiac arrest patients treated with targeted temperature management
Source: Crit Care. 2023 Mar 16;27:113. doi: 10.1186/s13054-023-04400-1 (PMC10022069; doi:10.1186/s13054-023-04400-1)
Supplement: Supplementary file 1 — Additional file 1. Additional statistical analysis of biomarkers for predicting poor neurological outcomes and comparison of novel and conventional biomarkers for predicting poor neurological outcomes. [file 13054_2023_4400_MOESM1_ESM.docx]

| **Supplementary table 1. AUCs of novel biomarkers for predicting poor neurological outcomes at 6 months after cardiac arrest** | | | | | | | |
| --- | --- | --- | --- | --- | --- | --- | --- |
| Biomarkers | Time | AUC | Cuttoff value | Sensitivity (95% CI) | Specificity (95% CI) | PPV | NPV |
| NSE | 0h | 0.726 | 41.72 | 61.1 (46.9 - 74.1) | 82.2 (67.9 - 92.0) | 80.5 (68.0 - 88.9) | 63.8 (55.1 - 71.7) |
|  | 24h | 0.881 | 30.50 | 90.0 (78.2 - 96.7) | 78.3 (63.6 - 89.1) | 81.8 (72.1 - 88.7) | 87.8 (75.6 - 94.4) |
|  | 48h | 0.860 | 34.60 | 75.6 (59.7 - 87.6) | 88.9 (75.9 - 96.3) | 86.1 (72.7 - 93.5) | 80.0 (69.8 - 87.4) |
|  | 72h | 0.857 | 30.18 | 72.2 (54.8 - 85.8) | 87.8 (73.8 - 95.9) | 83.9 (69.1 - 92.4) | 78.3 (67.7 - 86.1) |
|  | peak 0~72h | 0.873 | 54.80 | 85.2 (72.9 - 93.4) | 82.6 (68.6 - 92.2) | 85.2 (75.2 - 91.6) | 82.6 (71.2 - 90.1) |
|  | peak 24~72h | **0.890** | 54.60 | 78.0 (64.0 - 88.5) | 93.5 (82.1 - 98.6) | **92.9 (81.2 - 97.5)** | 79.6 (69.8 - 86.9) |
| S-100B | 0h | 0.699 | 0.87 | 59.3 (45.0 - 72.4) | 87.0 (73.7 - 95.1) | 84.2 (71.0 - 92.1) | 64.5 (56.4 - 71.9) |
|  | 24h | **0.901** | 0.18 | 79.0 (62.7 - 90.4) | 93.3 (77.9 - 99.2) | **93.7 (79.6 - 98.3)** | 77.8 (65.2 - 86.7) |
|  | 48h | 0.836 | 0.14 | 75.7 (57.7 - 88.9) | 86.7 (69.3 - 96.2) | 86.2 (71.1 - 94.1) | 76.5 (63.6 - 85.8) |
|  | 72h | 0.824 | 0.16 | 74.1 (53.7 - 88.9) | 80.7 (62.5 - 92.5) | 76.9 (61.1 - 87.6) | 78.1 (64.9 - 87.4) |
|  | peak 0~72h | 0.849 | 1.47 | 73.2 (57.1 - 85.8) | 93.6 (78.6 - 99.2) | **93.7 (79.5 - 98.3)** | 72.5 (61.2 - 81.5) |
|  | peak 24~72h | 0.866 | 0.26 | 73.5 (55.6 - 87.1) | 90.3 (74.2 - 98.0) | 89.3 (73.6 - 96.1) | 75.7 (63.7 - 84.6) |
| Tau | 0h | 0.724 | 1.79 | 85.7 (72.8 - 94.1) | 52.5 (36.1 - 68.5) | 68.9 (60.4 - 76.2) | 69.2 (56.4 - 79.7) |
|  | 24h | 0.767 | 1.07 | 89.5 (75.2 - 97.1) | 59.1 (43.2 - 73.7) | **93.7 (79.6 - 98.3)** | 77.8 (65.2 - 86.7) |
|  | 48h | 0.837 | 4.84 | 78.4 (61.8 - 90.2) | 83.3 (68.6 - 93.0) | 85.3 (71.5 - 93.1) | 82.2 (71.3 - 89.6) |
|  | 72h | **0.906** | 4.37 | 86.1 (70.5 - 95.3) | 88.4 (74.9 - 96.1) | 86.1 (72.9 - 93.5) | 88.4 (77.0 - 94.5) |
|  | peak 0~72h | 0.823 | 4.67 | 86.5 (74.2 - 94.4) | 68.2 (52.4 - 81.4) | 76.3 (67.3 - 83.4) | 81.1 (67.6 - 89.8) |
|  | peak 24~72h | 0.627 | 4.13 | 64.8 (50.6 - 77.3) | 78.3 (63.6 - 89.1) | 77.8 (66.8 - 85.9) | 89.5 (76.8 - 95.6) |
| NFL | 0h | 0.664 | 20.00 | 75.5 (61.1 - 86.7) | 52.5 (36.1 - 68.5) | 66.1 (57.5 - 73.7) | 63.6 (49.7 - 75.6) |
|  | 24h | 0.900 | 236.00 | 73.7 (56.9 - 86.6) | 93.2 (81.3 - 98.6) | 90.3 (75.5 - 96.6) | 80.4 (70.5 - 87.5) |
|  | 48h | 0.921 | 247.00 | 86.1 (70.5 - 95.3) | 88.1 (74.4 - 96.0) | 86.1 (72.9 - 93.4) | 88.1 (76.5 - 94.4) |
|  | 72h | **0.946** | 317.00 | 85.7 (69.7 - 95.2) | 93.0 (80.9 - 98.5) | 90.9 (76.9 - 96.8) | 88.9 (78.0 - 94.8) |
|  | peak 0~72h | 0.819 | 325.00 | 69.2 (54.9 - 81.3) | 86.4 (72.6 - 94.8) | 85.7 (73.6 - 92.8) | 80.4 (60.8 - 78.4) |
|  | peak 24~72h | 0.918 | 582.00 | 77.5 (61.5 - 89.2) | 95.5 (84.5 - 99.4) | **93.9 (79.8 - 98.4)** | 82.4 (72.3 - 89.3) |
| GFAP | 0h | 0.850 | 209.00 | 72.3 (57.4 - 84.4) | 85.0 (70.2 - 94.3) | 85.0 (72.6 - 92.4) | 72.3 (61.8 - 80.9) |
|  | 24h | 0.827 | 257.00 | 79.4 (62.1 - 91.3) | 79.6 (64.7 - 90.2) | 75.0 (62.0 - 84.6) | 83.3 (71.8 - 90.8) |
|  | 48h | 0.839 | 416.00 | 81.3 (63.6 - 92.8) | 78.6 (63.2 - 89.7) | 74.3 (61.3 - 84.1) | 84.6 (72.4 - 92.0) |
|  | 72h | 0.875 | 402.00 | 78.8 (61.1 - 91.0) | 90.7 (77.9 - 97.4) | **86.7 (71.5 - 94.4)** | 84.8 (74.1 - 91.5) |
|  | peak 0~72h | 0.860 | 474.00 | 80.0 (66.3 - 90.0) | 79.6 (64.7 - 90.2) | 81.6 (70.9 - 89.0) | 77.8 (66.3 - 86.1) |
|  | peak 24~72h | **0.881** | 846.00 | 77.8 (60.8 - 89.9) | 88.6 (75.4 - 96.2) | 84.8 (70.7 - 92.9) | 83.0 (72.4 - 90.1) |
| UCH-L1 | 0h | 0.766 | 99.88 | 77.6 (63.4 - 88.2) | 65.0 (48.3 - 79.4) | 73.1 (63.4 - 81.0) | 70.3 (57.3 - 80.7) |
|  | 24h | 0.886 | 54.52 | 84.6 (69.5 - 94.1) | 81.8 (67.3 - 91.8) | 80.5 (68.5 - 88.7) | 85.7 (73.9 - 92.7) |
|  | 48h | 0.911 | 120.00 | 83.8 (68.0 - 93.8) | 88.1 (74.4 - 96.0) | 86.1 (72.9 - 93.5) | 86.0 (74.6 - 92.8) |
|  | 72h | **0.935** | 76.30 | 86.1 (70.5 - 95.3) | 90.7 (77.9 - 97.4) | 88.6 (75.1 - 95.2) | 88.6 (77.5 - 94.7) |
|  | peak 0~72h | 0.893 | 145.50 | 92.3 (81.5 - 97.9) | 70.5 (54.8 - 83.2) | 78.7 (69.9 - 85.4) | 88.6 (74.8 - 95.3) |
|  | peak 24~72h | 0.913 | 191.00 | 82.5 (67.2 - 92.7) | 90.9 (78.3 - 97.5) | **89.2 (76.2 - 95.5)** | 85.1 (74.3 - 91.9) |
|  |  |  |  |  |  |  |  |

AUC=area under the curve; PPV=positive predictive value; NPV=negative predictive value; NSE=neuron specific enolase; S100-B= S100 calcium-binding protein; NFL=neurofilament light chain; GFAP=glial fibrillary acidic protein; UCHL1=ubiquitin C-terminal hydrolase-L1

**Supplementary table 2. P values of comparison of AUC curves of novel and conventional biomarkers for predicting poor neurological outcomes after cardiac arrest**

| 0hr | NSE | S100B |
| --- | --- | --- |
| Tau | 0.5856 | 0.9648 |
| NFL | 0.4068 | 0.8932 |
| GFAP | **0.0307** | **0.0029** |
| UCH-L1 | 0.5256 | 0.1967 |
| 24hr |  |  |
| Tau | 0.2669 | 0.2112 |
| NFL | 0.2409 | 0.8303 |
| GFAP | 0.9287 | 0.2238 |
| UCH-L1 | 0.4835 | 0.3966 |
| 48hr |  |  |
| Tau | 0.7898 | 0.4824 |
| NFL | 0.0685 | **0.0333** |
| GFAP | 0.6758 | 0.8499 |
| UCH-L1 | 0.2339 | **0.0135** |
| 72hr |  |  |
| Tau | 0.1260 | 0.2086 |
| NFL | **0.0168** | **0.0148** |
| GFAP | 0.2113 | 0.1214 |
| UCH-L1 | **0.0361** | **0.0069** |
